# Aspartate Aminotransferase to Platelet Ratio Index (APRI), Göteborg University Cirrhosis Index (GUCI), and Fibrosis 4 Score (FIB-4) proved to be the optimal biomarkers for the assessment of liver fibrosis in patients chronically infected with hepatitis C virus in Brazil

**DOI:** 10.1590/1806-9282.20250241

**Published:** 2025-09-19

**Authors:** Bianca Peixoto Dantas, Mariana Cavalheiro Magri, Caroline Manchiero, Arielle Karen da Silva Nunes, Thamiris Vaz Gago Prata, Fátima Mitiko Tengan

**Affiliations:** 1 Universidade de São Paulo, Hospital das Clinicas, Faculdade de Medicina, Laboratório de Investigação Médica em Hepatologia por Virus (LIM-47) – São Paulo (SP), Brazil.; 2 Universidade de São Paulo, Instituto de Medicina Tropical de São Paulo, Faculdade de Medicina – São Paulo (SP), Brazil.; 3 Universidade de São Paulo, Faculdade de Medicina, Departamento de Molestias Infecciosas e Parasitarias – São Paulo (SP), Brazil.

## INTRODUCTION

Hepatitis C infection is caused by hepatitis C virus (HCV) and is characterized by liver inflammation, being considered the leading cause of chronic liver disease^1^. An estimated 50 million people are infected with HCV, and 1.0 million new infections occur each year^2^. Notably, 10–20% of chronically infected patients can develop cirrhosis, liver failure, and hepatocellular carcinoma (HCC) over a period of 20–30 years^3^. The risk of progression from cirrhosis to HCC is approximately 1–6% per year^4^. Viral clearance reduces the risk of HCC but does not eliminate it. Hence, it is important to identify and monitor the patient's stage of liver disease^5^. It has been observed that, after treatment, fibrosis may be reversed^6,7^.

The anatomopathological study of a liver fragment (liver biopsy) is the gold standard for evaluating the fibrosis stage in patients with liver disease. However, it is an invasive method with some contraindications, and people living far from major centers may be deprived of this procedure. Noninvasive biomarkers may be used to determine the stage of fibrosis in these patients before treatment^1^.

The aim of this study was to define the optimal cutoff points for six noninvasive indirect biomarkers—namely, Aspartate Aminotransferase to Platelet Ratio Index^8^ (APRI), Fibrosis 4 Score^9^ (FIB-4), Forns Index^10^, Lok Index^11^, Göteborg University Cirrhosis Index^12^ (GUCI), and FibroIndex^13^—in individuals chronically infected with HCV from Brazil, by comparing these markers with liver biopsy data to assess fibrosis stage.

## METHODS

The present study was approved by the Ethics Committee for Analysis of Research Projects (CAPPesq) of the Clinical Hospital, School of Medicine, University of Sao Paulo (HCFMUSP), under protocol number 19230719.7.0000.0068 and approval number 3.644.473, on October 16, 2019. This retrospective study utilized data extracted from patients’ medical records, without direct intervention with the participants. Therefore, the Ethics Committee waived the need for informed consent.

This is a retrospective study that reviewed medical records of patients diagnosed with chronic HCV infection at HCFMUSP, Brazil, between January 2010 and December 2015. To select study participants, lists of patients who had been hospitalized or received outpatient care at the Division of Clinical Infectious and Parasitic Diseases at HCFMUSP were used. These lists were provided by the Administrative Data Section (medical files) in Excel spreadsheets, in which filters were applied to identify the patients of interest. For example, the International Classification of Diseases (ICD) code B18.2 was used to identify patients with chronic HCV infection.

The inclusion criteria were as follows: patients aged ≥18 years, diagnosed with chronic hepatitis C infection confirmed by the presence of HCV-RNA for more than 6 months, and who had undergone anatomopathological study of a liver fragment. The exclusion criteria were as follows: patients with hepatitis B virus or human immunodeficiency virus infection, decompensated liver disease, diagnosed with HCC, or who had undergone previous liver transplantation.

Demographic data and laboratory test results of alanine aminotransferase (ALT), aspartate aminotransferase (AST), gamma-glutamyl transferase (GGT), platelets, international normalized ratio (INR) derived from prothrombin time, cholesterol, and gamma-globulin were extracted from electronic medical records. There was an interval of up to 6 months between the liver biopsy and blood sample collection.

The diagnosis of HCV infection was performed by detecting the viral RNA using qualitative polymerase chain reaction (PCR). The liver biopsy was performed in all patients to stage the liver fibrosis according to the METAVIR scale, as follows: F0=absent fibrosis, F1=portal fibrosis without septa, F2=portal fibrosis with rare septa, F3=numerous septa without cirrhosis, and F4=cirrhosis^14^. Patients were analyzed in groups according to the fibrosis stage determined by the liver biopsy, as follows: significant fibrosis (F234), advanced fibrosis (F34), and those with cirrhosis (F4). We used the liver biopsy data collected before treatment. All liver biopsies were performed under the guidance of ultrasound equipment and were analyzed by the same medical team of the hospital, which is a reference center for anatomopathological evaluation in Brazil.

The diagnostic performance of noninvasive biomarkers was evaluated by sensitivity and specificity, as well as by building receiver operating characteristic (ROC) curves to measure the area under the receiver operating characteristic curve (AUROC) and to determine the optimal cutoff points. The AUROC was classified as described by Simundic^15^. The discriminative power of each biomarker was compared to data from the anatomopathological study of the liver fragment.

For the statistical analysis, continuous variables were expressed as mean values. Statistical significance was considered when p≤0.05. Statistical analysis was performed by using the MedCalc statistical software, v. 19.1.6.

## RESULTS

**Figure 1:**
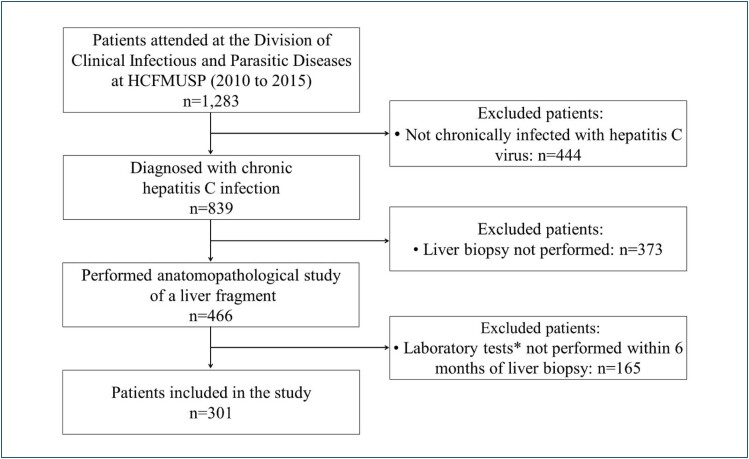
A flowchart depicting the selection of patients chronically infected with hepatitis C virus. HCFMUSP: Clinical Hospital, School of Medicine, University of Sao Paulo. *Alanine aminotransferase, aspartate aminotransferase, gamma-glutamyl transferase, platelets, international normalized ratio derived from prothrombin time, cholesterol, and gamma-globulin.

A flowchart depicting the selection of patients chronically infected with HCV is presented in Figure 1, and the demographic and laboratory characteristics of 301 retrospectively selected patients are shown in Table 1. Of these patients, 77.1% self-reported to be White, 15% pardo (mixed ethnic ancestry, also called Brown), 6.6% Black, 0.7% Yellow, and 0.7% did not report their racial/ethnic background. The mean age of the patients was 47 years, and 48.2% were female. As for liver biopsies, the median size of the fragment was 1.8 cm, ranging from 0.6 to 2.5 cm. Notably, 53% of the fragments were larger than 1.8 cm, whereas 21% measured between 1.6 and 1.7 cm.

**Table 1:** Demographic and laboratory characteristics of patients chronically infected with hepatitis C virus, according to liver fibrosis stage assessed with the METAVIR scale.

Characteristics	Fibrosis stage				
	F0 (n=46)	F1 (n=120)	F2 (n=74)	F3 (n=45)	F4 (n=16)
Age (years), mean±SD		42.2±10.3	45.7±11.6	47.8±11.6	53±11.6	53.5±10.2
**Sex**						
	Female, n (%)	22 (7.3)	62 (20.7)	37 (12.3)	17 (5.6)	7 (2.3)
	Male, n (%)	24 (7.9)	58 (19.3)	37 (12.3)	28 (9.3)	9 (3)
AST (U/L), median (IQR)		28 (19)	34 (23.5)	47.5 (34)	65 (58)	77 (52)
ALT (U/L), mean±SD		40.5±19.7	59±49.2	73.6±42.7	109.4±72.2	120.5±59.9
GGT (U/L), median (IQR)		39.5 (44)	43 (64)	79.5 (90)	86 (81)	88.5 (56)
INR, median (IQR)		1 (0.1)	1 (0.1)	1 (0.09)	1.06 (0.1)	1.1 (0.1)
Platelets, (mL/mm^3^), mean±SD		249±61.1	228±62.4	204±45.3	180±68.7	122±32
Cholesterol (mg/dL), mean±SD		172.4±37.02	166.6±33.08	172.5±36.6	151±35.7	148.9±23.9
Gamma-globulin (g/dL), median (IQR)		1.4 (0.3)	1.5 (0.4)	1.5 (0.5)	1.9 (0.7)	2.05 (0.6)

Mean±SD are presented for parameters with parametric distribution and median (IQR) for those with non-parametric distribution, tested by the Kolmogorov-Smirnov test of normality. AST: aspartate aminotransferase; ALT: alanine aminotransferase; GGT: gamma-glutamyl transferase; INR: international normalized ratio; IQR: interquartile range; SD: standard deviation.

### Performance of noninvasive indirect biomarkers

The performance of noninvasive biomarkers used to discriminate fibrotic stages—F0, F1, F2, F3, F4, F234, and F34—in patients chronically infected with HCV is detailed in Table 2. The biomarkers with AUROC values between 0.8 and <0.9 were classified as very good and those with AUROC values >0.9 as excellent, and are highlighted in bold. In the analysis of the diagnostic performance of noninvasive biomarkers in determining the presence of cirrhosis, FIB-4, FibroIndex, and Forns Index were classified as excellent, whereas APRI, GUCI, and Lok Index were classified as very good. For significant liver fibrosis (F34), APRI, FIB-4, GUCI, FibroIndex, and Forns Index were classified as very good, and in the case of advanced fibrosis (F234), APRI and GUCI were classified as very good.

**Table 2:** Diagnostic performance of noninvasive biomarkers in patients chronically infected with hepatitis C virus, according to liver fibrosis stages F0, F1, F2, F3, F4, F234, and F34, assessed by the METAVIR scale.

		APRI	FIB-4	FibroIndex	Forns Index	GUCI	Lok Index
**Absent fibrosis (F0)**							
	Cutoff point	≤0.6536	≤1.1321	≤1.3736	≤4.4154	≤0.6579	≤0.2144
	AUROC	0.786	0.764	0.750	0.734	0.786	0.681
	Sensitivity, %	93.5	80.4	93.48	65.22	93.5	60.9
	Specificity, %	50.2	65.9	54.90	73.33	51.8	69.8
**F1**							
	Cutoff point	≤0.6217	≤0.9757	≤1.9087	≤5.3375	≤0.6172	≤0.2106
	AUROC	0.659	0.654	0.626	0.633	0.660	0.585
**F2**							
	Cutoff point	>0.4252	>0.9757	>1.1541	>4.4189	>0.4252	>0.2125
	AUROC	0.616	0.594	0.552	0.548	0.616	0.537
**F3**							
	Cutoff point	>0.8832	>1.6933	>1.5954	>5.7598	>0.9078	>0.3491
	AUROC	0.765	0.754	0.751	0.761	0.766	0.655
	Sensitivity, %	66.7	66.7	75.56	75.56	66.7	55.6
	Specificity, %	77.7	72.7	72.27	68.36	78.1	72.7
**F4**							
	Cutoff point	>1.3215	>2.1628	>1.8494	>6.3609	>1.3215	>0.3063
	AUROC	**0.898**	**0.922**	**0.918**	**0.902**	**0.898**	**0.841**
	Sensitivity, %	93.7	93.7	93.75	93.75	93.7	93.7
	Specificity, %	86.7	83.5	82.11	75.79	86.7	62.1
**F34**							
	Cutoff point	>0.9304	>2.1628	>1.5954	>5.7598	>0.9078	>0.347
	AUROC	**0.833**	**0.831**	**0.828**	**0.831**	**0.833**	0.728
	Sensitivity, %	72.1	62.3	80.33	81.97	73.8	62.3
	Specificity, %	84.2	90	76.67	72.92	82.9	75.8
**F234**							
	Cutoff point	>0.6401	>1.1641	>1.3736	>5.3375	>0.6579	>0.2165
	AUROC	**0.804**	0.787	0.753	0.752	**0.805**	0.677
	Sensitivity, %	71.9	82.2	71.11	71.11	71.1	80
	Specificity, %	75.3	62.7	71.69	69.28	76.5	48.8

Very good (>8) to excellent (>9) AUROC values are in bold. AUROC: area under the ROC curve; F: fibrosis; APRI: Aspartate Aminotransferase to Platelet Ratio Index; FIB-4: Fibrosis 4 Score; GUCI: Göteborg University Cirrhosis Index.

### Comparison between noninvasive indirect biomarkers

In a comparison between the noninvasive indirect biomarkers for liver fibrosis stages, it was observed that in the group with cirrhosis (F4), all noninvasive biomarkers had AUROC values close to 0.9, with no statistically significant differences (p≥0.05). In the group with advanced fibrosis (F34), ROC curve analysis showed that APRI, FIB-4, FibroIndex, Forns Index, and GUCI had an AUROC value of 0.83. The Lok Index had a lower AUROC value, which was statistically significantly different from the other biomarkers (p<0.05). In the group with significant fibrosis (F234), ROC curve analysis showed that APRI, GUCI, and FIB-4 had AUROC values ranging from 0.79 to 0.80. Again, the Lok Index showed a lower AUROC value, which was statistically significantly different from the other biomarkers (p<0.05).

### Comparison of the cutoff points for noninvasive biomarkers

The comparison of the cutoff points for noninvasive biomarkers (APRI, FIB-4, and GUCI) in the present study and those described in the literature, both conducted on Brazilian patients chronically infected with HCV, is presented in Table 3. In this study, the proposed cutoffs demonstrated high sensitivity for cirrhosis (F4), exceeding 93%, with specificity exceeding 83%. In comparison, literature-based cutoffs showed a wider range of sensitivity (37–100%) and specificity (60–97%). For advanced fibrosis (F34), sensitivity exceeded 62% and specificity exceeded 83%, while literature cutoffs yielded a wider range of sensitivity (36–90%) and specificity (45–97%). For significant fibrosis (F234), the study cutoffs showed that sensitivity exceeded 71% and specificity exceeded 62%, while literature-based cutoffs yielded a wider range of sensitivity (19–82%) and specificity (57–96%).

**Table 3:** Sensitivity and specificity of Aspartate Aminotransferase to Platelet Ratio Index, Fibrosis 4 Score, and Göteborg University Cirrhosis Index with cutoffs stipulated in this study and those described in the literature, in the diagnosis of patients chronically infected with hepatitis C virus.

Noninvasive biomarker		Cutoffs in the present study	Sen %	Spe %	Cutoffs in the literature	Sen %	Spe %	References (literature)
**(F4)**								
	APRI	>1.3215	93.7	86.7	>1	93.7	78.2	Chou and Wasson^20^
					≥0.675	93.7	60.5	Cheng et al.^16^
					>1.30	93.7	86.5	Andrés-Otero et al.^18^
					>1.53	75	89.5	Itakura et al.^17^
					>2	50	92.9	Wai et al.^8^
	FIB-4	>2.1628	93.7	83.5	>1.45	100	61.4	Chou and Wasson^20^
					>2.63	75	90.2	Andrés-Otero et al.^18^
					>3.25	56.2	93.3	Chou and Wasson^20^
					>4.018	37.5	96.8	Cheng et al.^16^
					>4.32	37.5	97.2	Itakura et al.^17^
	GUCI	>1.3215	93.7	86.7	>1	93.7	77.5	Islam et al.^12^
					>1.15	93.7	82.8	Andrés-Otero et al.^18^
					>1.56	75	88.7	Chou and Wasson^20^
**(F34)**								
	APRI	>0.9304	72.1	84.2	>0.472	90.2	45.5	Cheng et al.^16^
					>0.77	73.7	76.7	Itakura et al.^17^
					>1.30	52.5	91	Andrés-Otero et al.^18^
	FIB-4	>2.1628	62.3	90	>3.25	36	97.5	Sterling et al.^9^
					>1.79	70.5	80.8	Cheng et al.^16^
					>3.26	36	97.5	Itakura et al.^17^
					>2.05	62.3	85.8	Andrés-Otero et al.^18^
	GUCI	>0.9078	73.8	82.9	>1.10	60.7	87.5	Andrés-Otero et al.^18^
**(F234)**								
	APRI	>0.6401	71.9	75.3	≥1.5	28.1	96.4	Wai et al.^8^
					≥0.5	80.7	60.2	Chou and Wasson^20^
					>0.48	82.2	57.2	Cordie et al.^19^
					>1.09	38	91.6	Andrés-Otero et al.^18^
	FIB-4	>1.1641	82.2	62.7	>1.45	65.2	77.1	Chou and Wasson^20^
					>3.25	19.3	98.8	Chou and Wasson^20^
					>1.27	72.6	67.5	Chou and Wasson^20^
					>1.50	60.7	79.5	Andrés-Otero et al.^18^
	GUCI	>0.6579	71.1	76.5	>0.57	77	66.3	Cordie et al.^19^
					>1.02	44.4	89.7	Andrés-Otero et al.^18^

(F4): cirrhosis; (F34): advanced fibrosis; (F234): significant fibrosis; Sen: sensitivity; Spe: specificity; APRI: Aspartate Aminotransferase to Platelet Ratio Index; FIB-4: Fibrosis 4 Score; GUCI: Göteborg University Cirrhosis Index.

## DISCUSSION

The identification and use of noninvasive biomarkers to assess liver fibrosis has been an important area of medical research, driven by the goal of improving patient care. The findings of this study highlight the optimal cutoff points for noninvasive biomarkers of liver fibrosis in patients chronically infected with HCV from HCFMUSP, Brazil. Among the biomarkers evaluated—APRI, FIB-4, Forns Index, Lok Index, GUCI, and FibroIndex—APRI, GUCI, and FIB-4 presented the best diagnostic performance, demonstrating the highest AUROCs and the optimal cutoff points for the detection of significant fibrosis (F234), advanced fibrosis (F34), and cirrhosis (F4).

In the present study, the performance of FIB-4 and APRI in the diagnosis of F34 and cirrhosis was similar to that found in studies by Cheng et al.^16^, Itakura et al.^17^, and Andrés-Otero et al.^18^. In a Chinese study^16^, which evaluated the presence of F34 in 113 patients chronically infected with HCV, the authors obtained an AUROC value of 0.891 and a cutoff point ≥0.472 by APRI and an AUROC of 0.851 and a cutoff point ≥1.799 by FIB-4. Additionally, in the diagnosis of cirrhosis, an AUROC of 0.800 and a cutoff point ≥0.675 by APRI and an AUROC of 0.879 and a cutoff point ≥4.018 by FIB-4 were obtained.

In a Japanese study^17^, the AUROC values and cutoff points for APRI and FIB-4 in the diagnosis of F34 in patients with HCV were 0.781 and 0.77 and 0.796 and 3.26, respectively. In the case of cirrhosis, the AUROC values and cutoff points for APRI and FIB-4 were 0.824 and 1.53 and 0.852 and 4.32, respectively. A Spanish study^18^ compared 14 noninvasive biomarkers (of which five are evaluated in the present study, except Lok Index) in 83 patients with HCV. In their study, FIB-4 had the best diagnostic performance with excellent AUROC values for F34 and F4, with their respective AUROC values and cutoff points being 0.907 and 2.05 and 0.911 and 2.63. In the present study, in the diagnosis of F34 and F4, the five noninvasive biomarkers had AUROC values >0.8, and hence classified as very good.

Regarding the diagnosis of F234, a study conducted in Egypt by Cordie et al.^19^ also found that FIB-4 (AUROC=0.783; cutoff point >1.27) had better performance compared to APRI (0.717; >0.48) and GUCI (0.723; >0.57). Moreover, a systematic review by Chou et al. that included 172 studies evaluated the diagnostic accuracy of blood tests and noninvasive biomarkers. They obtained an AUROC value >0.7 in the evaluation of F234 with platelet count, age–platelet index, APRI, FibroIndex, Fibrotest, and Forns index. In the presence of cirrhosis, the AUROC was >0.8 for platelet count, age–platelet index, APRI, and Hepascore^20^.

Diagnosing the presence/absence of cirrhosis (F4) is useful to determine the treatment management and to follow up these patients for the presence of varicose veins, liver failure, and HCC^21^. Noninvasive tests are usually cheaper, less risky, and more accessible^22^. The use of noninvasive tests, such as FIB-4, APRI, or elastography, was recommended by the World Health Organization for patients chronically infected with HCV in low- and middle-income resource settings^23^. Therefore, greater attention is needed to improve the accuracy of noninvasive biomarkers and consequently the diagnosis of liver fibrosis in low- and middle-income resource regions^24^.

In this study, on analyzing the original cutoff points, APRI, GUCI, and FIB-4 proved to be the best noninvasive biomarkers for the diagnosis of cirrhosis, advanced fibrosis, and significant fibrosis, respectively. We observed that the APRI and FIB-4 markers used cutoff points with specificity above 90%, and, therefore, the sensitivity was extremely low, around 30%. In the analysis of cirrhosis, we established a cutoff point with specificity >80% and sensitivity >90%. In the analysis of advanced fibrosis using FIB-4, we used the original cutoff point with a sensitivity of 36% and a specificity of 97% and a new cutoff point with a sensitivity of 62% and a specificity of 90%. When the cutoff points were adjusted for the diagnosis of significant fibrosis using APRI, an increased sensitivity from 28 to 71% and a decreased specificity from 96 to 75% were observed. In the analysis of cirrhosis, the original and new cutoff points for GUCI marker were similar. In the present study, for advanced and significant fibrosis, we established cutoff points with sensitivity and specificity greater than 70%.

The present study has the limitations of being retrospective and obtaining data from electronic medical records. Although our results were not very different from those found elsewhere, we believe that the cutoff points we detected in the ROC curves to differentiate the main groups in clinical practice should serve as a reference in the management of patients, especially in populations from Brazilian areas with limited resources, for whom liver biopsy and imaging tests are inaccessible and/or contraindicated.

## CONCLUSION

It can be said that the optimal cutoff points for six noninvasive indirect biomarkers (APRI, FIB-4, Forns Index, Lok Index, GUCI, and FibroIndex) used to assess liver fibrosis stages established in Brazilian patients chronically infected with HCV differed from those observed in some populations from other countries. Among these biomarkers, APRI, GUCI, and FIB-4 were the best in determining the significant fibrotic, advanced fibrotic, and cirrhotic stages of liver fibrosis in this group of patients.

## Data Availability

The datasets generated and/or analyzed during the current study are available from the corresponding author upon reasonable request.

## References

[1] European Association for the Study of the Liver (2020). EASL recommendations on treatment of hepatitis C: final update of the series. J Hepatol.

[2] World Health Organization (2024). Hepatitis C.

[3] Roudot-Thoraval F (2021). Epidemiology of hepatitis C virus infection. Clin Res Hepatol Gastroenterol.

[4] Llovet JM, Kelley RK, Villanueva A, Singal AG, Pikarsky E, Roayaie S (2021). Hepatocellular carcinoma. Nat Rev Dis Primers.

[5] Kanwal F, Kramer JR, Asch SM, Cao Y, Li L, El-Serag HB (2020). Long-term risk of hepatocellular carcinoma in HCV patients treated with direct acting antiviral agents. Hepatology.

[6] Caligiuri A, Gentilini A, Pastore M, Gitto S, Marra F (2021). Cellular and molecular mechanisms underlying liver fibrosis regression. Cells.

[7] Pei Q, Yi Q, Tang L (2023). Liver fibrosis resolution: from molecular mechanisms to therapeutic opportunities. Int J Mol Sci.

[8] Wai CT, Greenson JK, Fontana RJ, Kalbfleisch JD, Marrero JA, Conjeevaram HS (2003). A simple noninvasive index can predict both significant fibrosis and cirrhosis in patients with chronic hepatitis C. Hepatology.

[9] Sterling RK, Lissen E, Clumeck N, Sola R, Correa MC, Montaner J (2006). Development of a simple noninvasive index to predict significant fibrosis in patients with HIV/HCV coinfection. Hepatology.

[10] Forns X, Ampurdanès S, Llovet JM, Aponte J, Quintó L, Martínez-Bauer E (2002). Identification of chronic hepatitis C patients without hepatic fibrosis by a simple predictive model. Hepatology.

[11] Lok AS, Ghany MG, Goodman ZD, Wright EC, Everson GT, Sterling RK (2005). Predicting cirrhosis in patients with hepatitis C based on standard laboratory tests: results of the HALT-C cohort. Hepatology.

[12] Islam S, Antonsson L, Westin J, Lagging M (2005). Cirrhosis in hepatitis C virus-infected patients can be excluded using an index of standard biochemical serum markers. Scand J Gastroenterol.

[13] Koda M, Matunaga Y, Kawakami M, Kishimoto Y, Suou T, Murawaki Y (2007). FibroIndex, a practical index for predicting significant fibrosis in patients with chronic hepatitis C. Hepatology.

[14] The French METAVIR Cooperative Study Group (1994). Intraobserver and interobserver variations in liver biopsy interpretation in patients with chronic hepatitis C. Hepatology.

[15] Šimundić AM (2009). Measures of diagnostic accuracy: basic definitions. EJIFCC.

[16] Cheng CH, Chu CY, Chen HL, Lin IT, Wu CH, Lee YK (2020). Subgroup analysis of the predictive ability of aspartate aminotransferase to platelet ratio index (APRI) and fibrosis-4 (FIB-4) for assessing hepatic fibrosis among patients with chronic hepatitis C. J Microbiol Immunol Infect.

[17] Itakura J, Kurosaki M, Setoyama H, Simakami T, Oza N, Korenaga M (2021). Applicability of APRI and FIB-4 as a transition indicator of liver fibrosis in patients with chronic viral hepatitis. J Gastroenterol.

[18] Andrés-Otero MJ, De-Blas-Giral I, Puente-Lanzarote JJ, Serrano-Aulló T, Morandeira MJ, Lorente S (2016). Multiple approaches to assess fourteen non-invasive serum indexes for the diagnosis of liver fibrosis in chronic hepatitis C patients. Clin Biochem.

[19] Cordie A, Salama A, El-Sharkawy M, El-Nahaas SM, Khairy M, Elsharkawy A (2018). Comparing the efficiency of Fib-4, Egy-score, APRI, and GUCI in liver fibrosis staging in Egyptians with chronic hepatitis C. J Med Virol.

[20] Chou R, Wasson N (2013). Blood tests to diagnose fibrosis or cirrhosis in patients with chronic hepatitis C virus infection: a systematic review. Ann Intern Med.

[21] Trivedi HD, Patwardhan VR, Malik R (2019). Chronic hepatitis C infection - noninvasive assessment of liver fibrosis in the era of direct acting antivirals. Dig Liver Dis.

[22] Chadha N, Sterling RK (2024). A clinical review of noninvasive tests for hepatic fibrosis. Gastroenterol Hepatol (N Y).

[23] World Health Organization (2024). Global hepatitis report 2024: action for access in low- and middle-income countries.

[24] Rungta S, Kumari S, Deep A, Verma K, Swaroop S (2021). APRI and FIB-4 performance to assess liver fibrosis against predefined Fibroscan values in chronic hepatitis C virus infection. J Family Med Prim Care.

